# Disseminated Varicella-Zoster Virus Infection Complicated by Encephalitis and Ramsay Hunt Syndrome in an HIV Patient

**DOI:** 10.7759/cureus.9235

**Published:** 2020-07-17

**Authors:** Amro Elshereye, Burak Erdinc, Sonu Sahni

**Affiliations:** 1 Internal Medicine, Brookdale University Hospital and Medical Center, Brooklyn, USA

**Keywords:** varicella zoster virus, ramsay hunt syndrome, encephalitis, hiv

## Abstract

Varicella-zoster virus (VZV) is a human α-herpesvirus which cause primary varicella infection (chicken pox) or herpes zoster infection (shingles) after reactivation of the dormant virus. VZV infection is usually self-limited but disseminated infection can be seen in immunocompromised individuals. It can also get complicated by central nervous system (CNS) involvement. We describe a case of a 51-year-old male with human immunodeficiency virus (HIV) who presented with altered mental status and deficits in his right-sided cranial nerves of VI, VII, and VIII. The patient also had disseminated vesicular-pustular rash all over his body at different stages of healing. A diagnosis of disseminated VZV infection complicated by encephalitis and Ramsay Hunt syndrome was made and the patient was treated with intravenous acyclovir and oral prednisone with a rapid improvement. The coexistence of these conditions is rare. The purpose of this report is to increase awareness of the coexistence of these two conditions in HIV infected patients.

## Introduction

Varicella-zoster virus (VZV) is a human α-herpesvirus. It can cause primary varicella infection (chicken pox) or herpes zoster infection (shingles) after reactivation of the dormant virus. VZV infection is usually self-limited but can be disseminated in immunocompromised individuals such as human immunodeficiency virus (HIV) infected patients. It can lead to widespread skin involvement and encephalitis [1]. Immunocompromised patients including patients with HIV or transplant recipients are at risk of VZV reactivation due to reduced T-cell mediated immunity [2]. Central nervous system (CNS) complications of VZV include myelitis, encephalitis, aseptic meningitis, acute cerebellar ataxia, Ramsay Hunt syndrome, and stroke [3]. Ramsay Hunt syndrome is a rare complication of VZV infection. It is characterized by VZV reactivation in the geniculate ganglion which can give a variable presentation characterized by ipsilateral facial paralysis, ear pain, and vesicles in the auditory canal [4,5]. We present a rare case of disseminated VZV with classical and demonstrative skin lesions in an HIV patient which was complicated by VZV encephalitis and Ramsay Hunt syndrome.

## Case presentation

**Figure 1:**
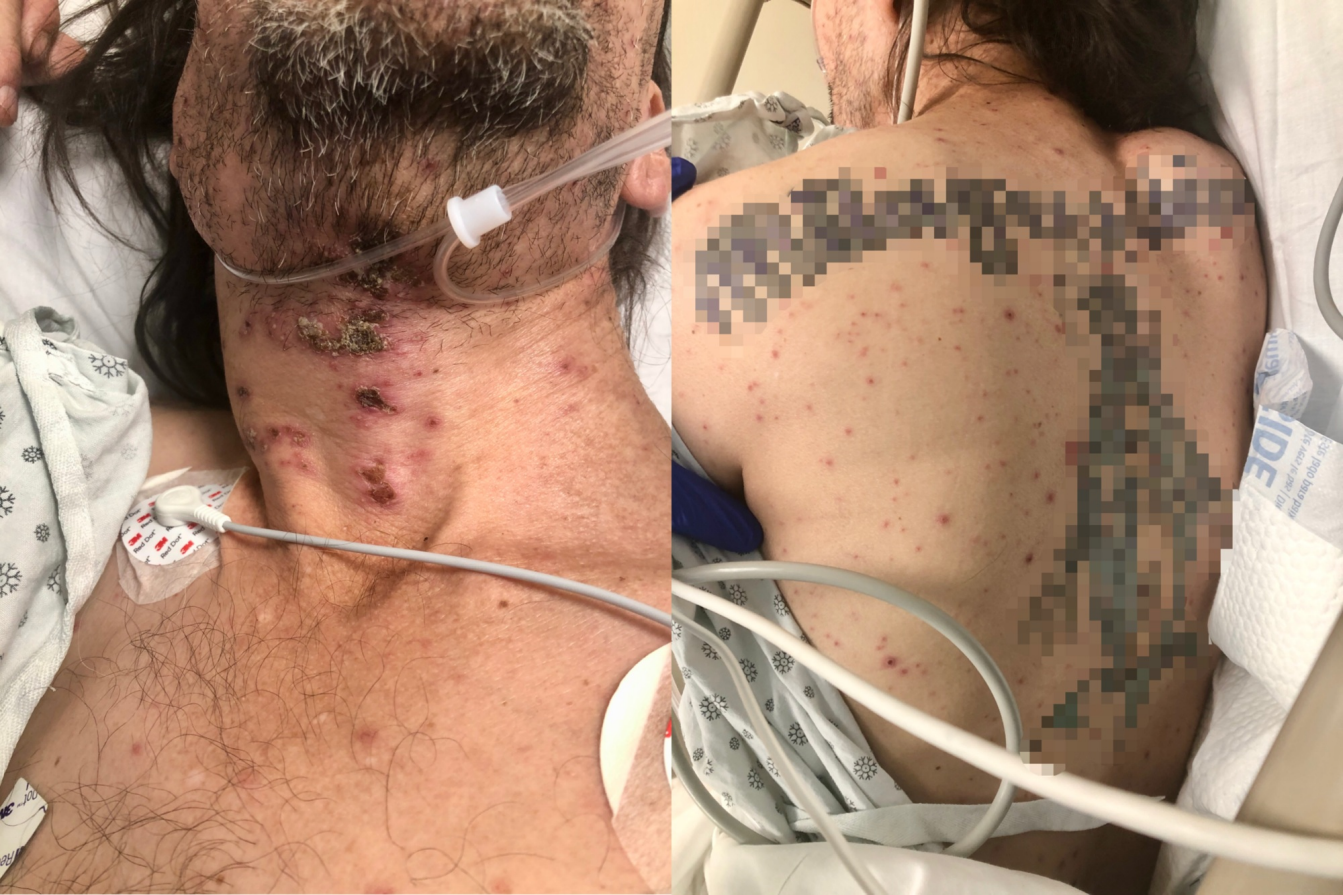
Vesicular-pustular rash in different stages of healing seen on the neck (left) and back (right). Patient's tattoos are censored for anonymity.

**Figure 2:**
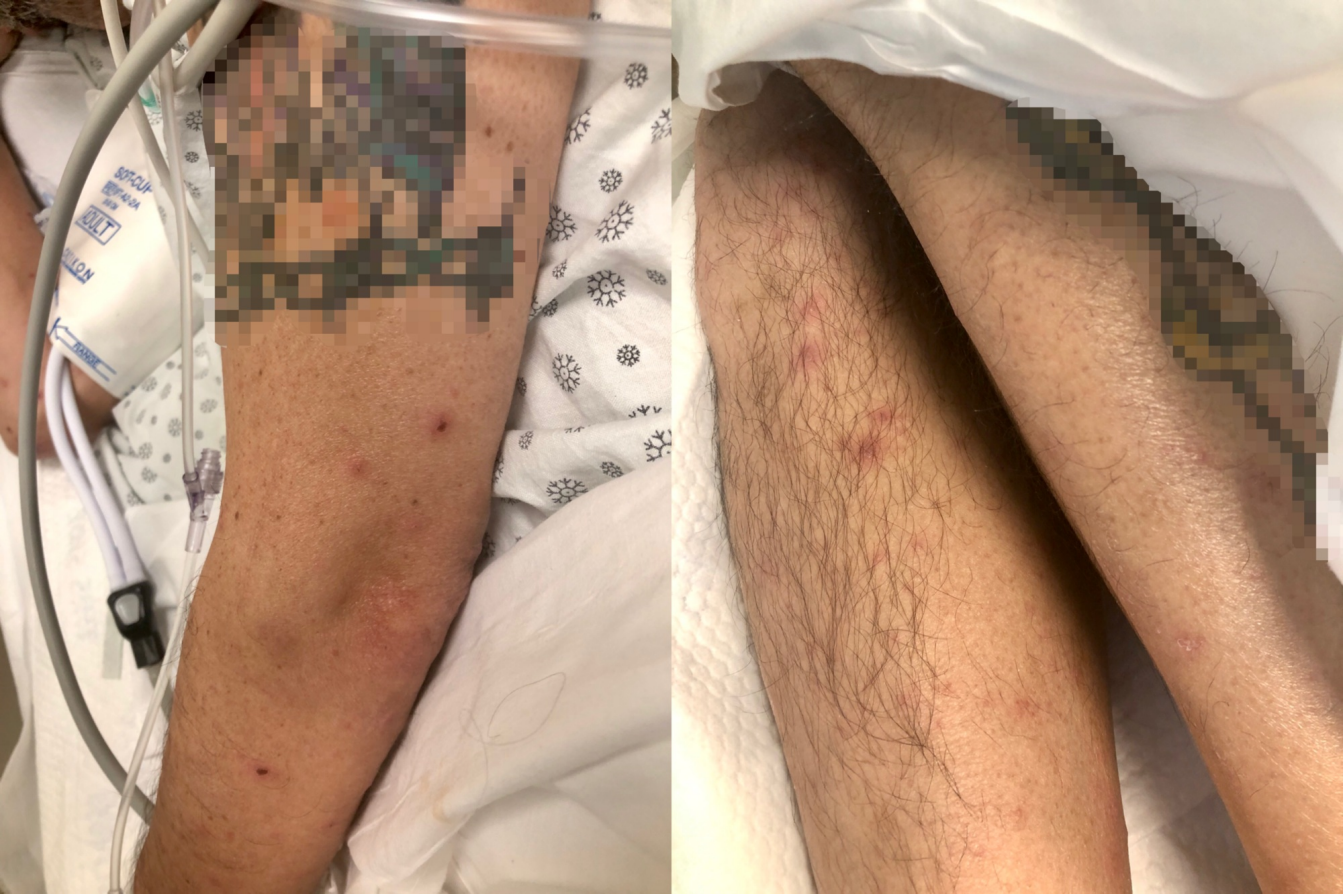
Vesicular-pustular rash in different stages of healing seen on the left arm (left) and both legs (right). Patient's tattoos are censored for anonymity.

A 50-year-old male who had a past medical history of HIV with unknown cluster of differentiation 4 (CD4) lymphocyte count and not compliant with his medications was brought in by emergency medical services (EMS) after he was found in his room covered in feces and looking disheveled. They brought him to Brookdale University Hospital and Medical Center emergency room. His initial vital signs showed a temperature of 100 ºF, blood pressure of 143/91 mmHg, heart rate of 105 beats per minute, respiratory rate of 20 per minute. He was noticed to have disseminated, vesicular-pustular rash all over his body at different stages of healing and crusted skin eruption on his anterior neck and submandibular area (Figures 1-2).

Patient’s neurological exam revealed that he was alert and oriented to self only, had a right-sided upper and lower facial droop. The patient also had impaired hearing on the right side. The muscle strength was 5/5 throughout. The patient had normal reflexes and the sensation was intact. The remainder of his physical exam did not show any other abnormal findings. Computerized tomography of the head without contrast showed no acute intracranial pathology. Subsequently, the patient had a lumbar puncture done in the emergency room which revealed a cloudy cerebrospinal fluid (CSF) with a white blood cell count of 849 cells/uL with lymphocyte predominance (72%), glucose of 39 mg/dl and protein of 364 mg/dl. Serum electrolytes, liver enzymes, and coagulation profile were all within normal limits. CSF sample was sent for a varicella-zoster polymerase chain reaction (PCR) test as well. His chest X-ray was clear without any infiltrates. His initial laboratory investigations are summarized in Table 1. 

**Table 1:** Initial laboratory investigations. NA: Not applicable

Complete blood count	Result	Reference values
White blood cell	11.90 x 10^3 ^/uL	4.10-10.10 10^3^/uL
Neutrophils	84.4%	44.5 - 73.4%
Lymphocytes	8.0%	17.8 - 42.0%
Monocytes	7.3%	5.7 - 11.2%
Hemoglobin	13.6 g/dl	12.9-16.7 g/dl
Platelets	249 x10^3^/uL	153 – 328 10x^3^/uL
Chemistry		
Blood urea nitrogen	27 mg/dL	9 – 20 mg/dL
Creatinine	0.63 mg/dL	0.66 – 1.25 mg/dL
Sodium	129 mEq	133 – 145 mEq/L
Potatssium	5.2 mEq/L	3.5 – 5.1 mEq/L
Bicarbonate	21 mEq/L	22- 30 mEq/L
Lactate	2.0 mmol/L	0.70 – 2.10 mmol/L
Cerebospinal fluid		
Appearance	Hazy. Cloudy	Clear
White blood cell	849 cells/uL	0.0 – 5.0 cells/uL
Red blood cell	25 cells/uL	No RBC’s cells/uL
Neutrophils	24%	NA
Lymphocytes	72%	NA
Macrophages	4%	NA

**Figure 3:**
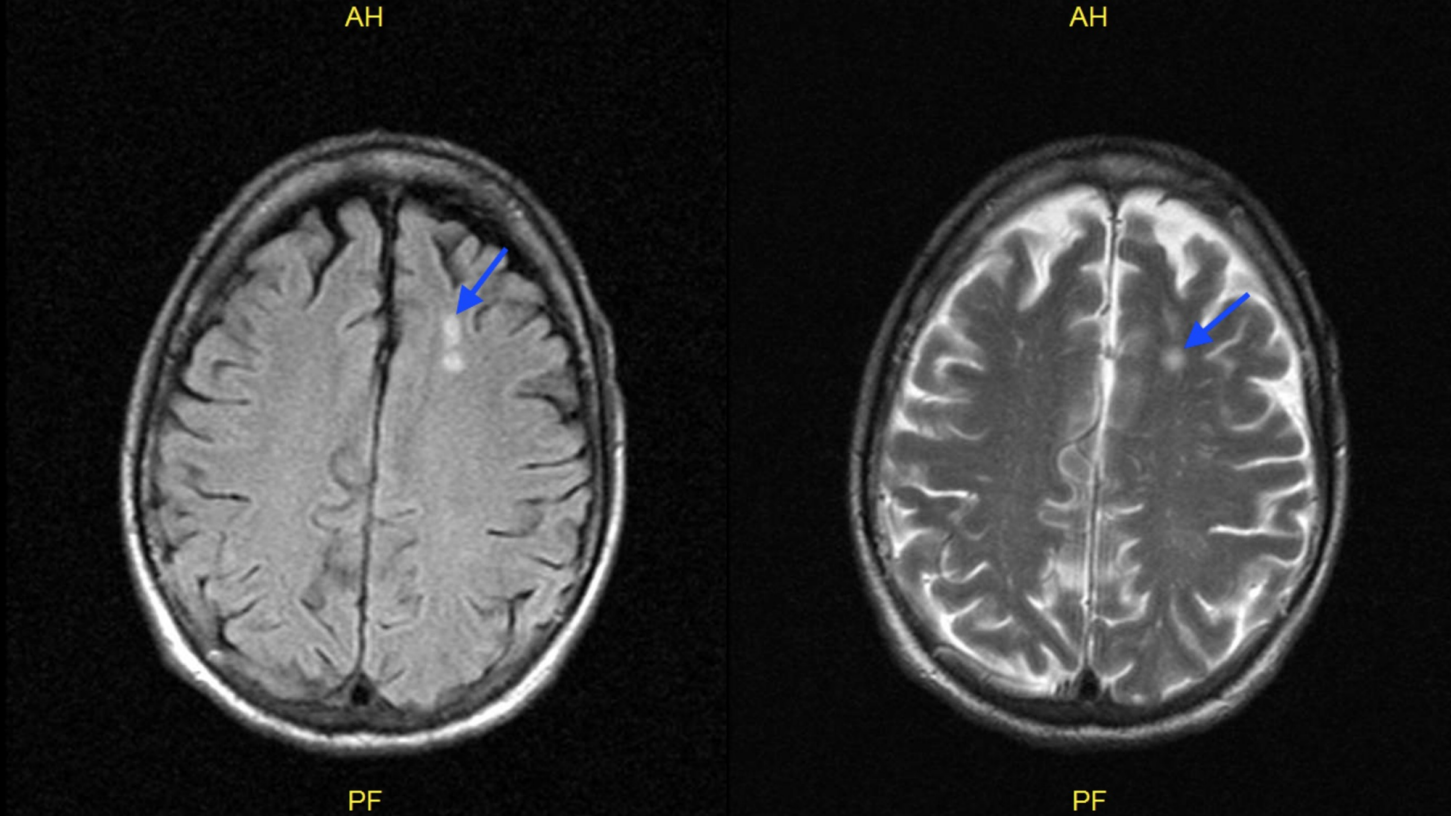
MRI brain without contrast showing nonspecific white matter lesions in the high left frontal lobe measuring approximately 8 mm (blue arrow).

A presumptive diagnosis of disseminated VZV with encephalitis was made according to the patient's skin lesions and CSF analysis. Therefore, he was started on intravenous acyclovir at 10 mg/kg every eight hours and admitted to the general medical floor under contact and airborne precautions. Patient’s varicella zoster PCR from the CSF sample came back positive. Patient mental status improved rapidly within 48 hours of treatment and he became alert and fully oriented. On eye exam, the patient had diplopia with right gaze, and it was determined that he had a right sixth cranial nerve palsy. He had deficits in right sided cranial nerves VI, VII, and VIII evident by impaired right eye abduction, right sided facial weakness, as well as impaired hearing. This constellation of findings is likely secondary to Ramsay Hunt syndrome with multiple cranial nerve involvement. Intravenous acyclovir was continued, and the patient started taking prednisone 50 mg orally for five days for the treatment of Ramsay Hunt syndrome. He was also found to have otitis externa and was prescribed ciprofloxacin with dexamethasone otic drops. Magnetic resonance imaging of the brain without contrast found nonspecific white matter lesions in the high left frontal lobe measuring approximately 8 mm but otherwise no mass, hemorrhage, or acute infarct (Figure 3).

The patient’s mental status came back to his baseline on day three. The patient’s rash had crusted on day six hence the isolation precautions were discontinued. He started ambulating without any difficulty and facial paralysis resolved by day eight. He was safely discharged home after nine days of hospital stay with oral acyclovir 400 mg tablets three times daily for 21 days and prednisone 50 mg tablets daily for three days.

## Discussion

Ramsay Hunt syndrome was first described by James Ramsay Hunt in 1907 in a patient who had ear pain and a vesicular rash. It was postulated at that time that it is the result of reactivation of latent VZV in the geniculate ganglion [4]. This hypothesis was confirmed by detecting varicella-zoster DNA by PCR in the geniculate ganglion [6]. This explains the facial nerve symptoms like facial droop but does not explain otologic symptoms such as hearing loss. It was later found that herpes zoster can remain dormant in the spiral and vestibular ganglia and its reactivation there might be the cause of the otologic symptoms [7].

Herpes encephalitis is a rare complication of VZV infection with an incidence of only 0.1%-0.2% in patients infected with VZV [8]. The encephalitis can precede the herpes zoster exanthem with up to 30 days or happen within 10 months after that but usually, it occurs within two weeks of the disseminated skin lesions as seen with our patient [1]. PCR is an excellent diagnostic modality for herpes encephalitis with over 95% sensitivity and specificity for detecting VZV in the CSF [9].

On review of the literature, an interesting observation was made that patients with VZV encephalitis CNS symptoms improve within 72 hours of acyclovir treatment compared to 14 days in patients who do not receive treatment [10]. This highlights the importance of aggressive early treatment of this entity with intravenous acyclovir [11]. The dosage is usually 30 mg/kg per day dosed three times daily. Ramsay Hunt syndrome is also treated with intravenous acyclovir combined with prednisone. Approximately 75% of patients treated with intravenous acyclovir recover from the facial paralysis in comparison to only 30% of patients who did not receive acyclovir [12]. Our patient regained facial nerve function which confirms the effectiveness of acyclovir in the treatment of Ramsay Hunt syndrome.

## Conclusions

HIV and other immunocompromised states can predispose patients to disseminated VZV infection including Ramsay Hunt syndrome and VZV encephalitis. The co-existence of Ramsay Hunt syndrome and varicella-zoster encephalitis is rare. It has not been described in the literature frequently and thus highlighting the diagnosis and treatment of such an entity is imperative especially due to the fact that outcomes are excellent with early aggressive treatment as seen with our patient.
